# Effectiveness of sodium bicarbonate combined with hydrogen peroxide and CPP-ACPF in whitening and microhardness of enamel

**DOI:** 10.4317/jced.53108

**Published:** 2017-03-01

**Authors:** Farzaneh Ahrari, Nadia Hasanzadeh, Omid Rajabi, Zakiyeh Forouzannejad

**Affiliations:** 1 DDS, MS, Dental Research Center, School of Dentistry, Mashhad University of Medical Sciences, Mashhad, Iran; 2 MD, Department of Drug Control, School of Pharmacy, Mashhad University of Medical Sciences, Mashhad, Iran; 3 DDS, Private Practice, Bushehr, Iran

## Abstract

**Background:**

This study investigated the effects of sodium bicarbonate (NaHCO3) combined with 1.5% hydrogen peroxide (H2O2) and casein phosphopeptide amorphous calcium phosphate fluoride (CPP-ACPF) on color and microhardness of enamel.

**Material and Methods:**

Seventy-five bovine incisors were immersed in a tea solution for 7.5 days. The specimens were randomly divided into five groups according to the whitening agent applied: 1) 94% NaHCO3, 2) a blend of 94% NaHCO3 and CPP-ACPF, 3) a blend of 94% NaHCO3 and 1.5% H2O2, 4) a blend of 94% NaHCO3, 1.5% H2O2 and CPP-ACPF, 5) control. The whitening procedure was performed for 10 times over 10 days. At each day, the buccal surfaces were covered with whitening agents for 5 minutes and then brushed for 30 seconds. After the 10 days, the teeth were again immersed in a tea solution for 10 minutes. Color assessment was performed at baseline (T1), after the first staining process (T2), after the whitening procedure (T3), and after the second staining process (T4). Finally, the specimens were subjected to microhardness test.

**Results:**

There was a statistically significant difference in the color change between T2 and T3 stages among the study groups (*p*<0.05), with the greatest improvement observed in group 4. Microhardness was significantly greater in groups 2 and 4, as compared to the other groups (*p*<0.05).

**Conclusions:**

The combination of 94% NaHCO3, 1.5% H2O2 and CPP-ACPF was effective in improving color and microhardness of teeth with extrinsic stains and could be recommended in the clinical situation.

## Introduction

A more esthetic and pleasant smile has been a common desire for most patients seeking dental treatments. Tooth color is generally considered as a main factor in dental attractiveness, particularly in the anterior region of upper dentition. Discoloration of the teeth may be resulted from extrinsic or intrinsic stains. Intrinsic stains are generated by endogenic chromogens within the enamel and dentin, whereas extrinsic stains are caused by the binding of exogenous chromogens to the enamel surfaces (1). Several methods have been proposed to remove discolorations including microabrasion, macroabrasion and bleaching. In recent years, the popularity of whitening agents that can be used by patients to lighten teeth has been increased. These products may be extremely useful in subjects suffering from extrinsic stains such as smokers and those undergoing fixed orthodontic therapy, as the placement of appliances could lead to a considerable amount of discoloration within a few days later.

Whitening products generally incorporate abrasive materials such as sodium bicarbonate (NaHCO3) in association with or without a mild bleaching component. The bleaching component, either hydrogen or carbamide peroxide, can remove extrinsic and intrinsic stains through oxidative mechanisms. An ideal whitener should eliminate surface deposits and stains with minimal influences on the properties of tooth enamel and restorations. However, it has been demonstrated that dentifrices containing whitening agents and abrasives could produce high levels of calcium release rates and enamel morphological lesions (2,3). Furthermore, tooth sensitivity and demineralization of dental structure due to the low PH of some bleaching agents have been reported as common side effects of tooth bleaching (4-8). Therefore, the use of a remineralizing agent such as casein phosphopeptide-amorphous calcium phosphate (CPP-ACP) has been recommended before, during or after the whitening process. It is believed that the application of CPP-ACP, a rich reservoir of bioavailable Ca and P ions can result in rapid mineral deposition on enamel crystallites and dentinal tubules, thus decreasing tooth sensitivity caused by the whitening products and enhancing remineralization (9-15). Furthermore, Singh *et al.* (16) reported that treatment of freshly bleached enamel with CPP-ACP or fluoride can significantly reduce further stain absorption compared to teeth without surface treatment.

In recent years, Tooth Mousse Plus (MI Paste Plus; GC Corporation, Tokyo, Japan) has been introduced into the market. This product combines CPP-ACP and 900 ppm fluoride (CPP-ACPF), and is assumed to provide more therapeutic effects than Tooth Mousse (MI Paste), which contains CPP-ACP alone (17-20). There is little information regarding the efficacy of abrasive and mild bleaching agents combined with a CPP-ACPF paste on removal of enamel stains, increasing mineral properties of enamel, and preventing further stain absorption. Therefore, the present study aimed to evaluate the effects of sodium bicarbonate blended with a low concentration of hydrogen peroxide and/or a CPP-ACPF paste on color change and microhardness of bovine enamel with extrinsic stains.

## Material and Methods

Seventy-five freshly extracted bovine incisors were selected and stored for 1 week in a 0.1% thymol solution. The teeth with visible caries, cracks or hypoplastic defects were excluded. The specimens were polished with water slurry of pumice and rubber prophylactic cups at low speed, and then stored in saline solution until preparation for testing.

The sample size for each group was calculated as n = 13, based on an alpha significance level of 0.05 and a beta of 0.1, according to the data obtained from a previous study (1) in which the mean ± standard deviation of one group (94% NaHCO3+1.5% H2O2) was 1.05 ± 0.85 and that of the control group (water) was 0.09 ± 0.55. This gave a power of 90 per cent to detect a significant difference in color change between group 1 and group 2 using a two-group t-test in NCSS/PASS software (NCSS Statistical Software, Kaysville, Utah). The sample size was then rounded up to 15.

-Preparation of the specimens

The roots of the teeth were sectioned 2 mm apically to the cemento-enamel junction using diamond disks. The crowns were then positioned in plastic molds and embedded in self-curing epoxy resin. The enamel surfaces of the teeth were ground flat using fine sandpaper disks. Grinding was continued until an enamel area measuring 6 mm in diameter was exposed in order to match the diameter of the spectrophotometer.

Afterwards, the specimens underwent an artificial staining procedure using a tea solution. The tea solution was prepared by boiling 2 g of tea in 100 ml of distilled water for 5 minutes. This solution was filtered to separate the tea from the infusion. Each crown was immersed in 10 ml of the staining solution for 7.5 days. A fresh tea solution was prepared every day throughout the staining period. The specimens were then rinsed thoroughly with distilled water and dried.

-The whitening procedure

After staining, the specimens were randomly divided into 5 groups of 15 and exposed to the whitening treatment. The whitening agents employed in the study groups were as follows:

Group 1: 94% NaHCO3 only

Group 2: a blend of 94% NaHCO3 and a CPP-ACPF paste

Group 3: a blend of 94% NaHCO3 and 1.5% H2O2 

Group 4: a blend of 94% NaHCO3, 1.5% H2O2 and a CPP-ACPF paste 

Group 5: distilled water as negative control 

The whitening agents in groups 1 to 4 were prepared in the form of paste in the Research Laboratory, School of Pharmacy, Mash-had University of Medical Sciences, Mashhad, Iran.

The whitening procedure was performed for 10 times over 10 days. At each day, the enamel surfaces were dried and covered by a 1 mm-thick layer of the whitening agent for 5 minutes. After that, the surfaces were manually cleaned by an electric toothbrush (Oral BVitality Floss Action, Germany) for 30 seconds and rinsed with distilled water. This procedure was repeated 10 times with 24-hour intervals. Between the whitening sessions, the samples were stored in daily replenished Fusayama Meyer artificial saliva at 37˚C. No whitening agent was applied in the control group and enamel surfaces were just subjected to mechanical abrasion by the electric toothbrush.

Twenty-four hours after the last whitening treatment, the specimens were removed from artificial saliva and once again immersed in a freshly prepared tea solution for 10 minutes in order to determine the susceptibility of the treated surfaces to further stain absorption.

-Color assessment

The color of the specimens was measured using an EasyShade spectrophotometer (Vita Zahnfabrik, Bad Säckingen, Germany) at four time points over the experiment: baseline (T1), after the 7.5-day staining process (T2), after the whitening procedure (T3), and after the second staining process (T4). Before obtaining the color measurements, the teeth were rinsed thoroughly with distilled water and allowed to air dry for 30 minutes. The tip of the spectrophotometer was placed at the 6-mm diameter aperture over the central part of the buccal enamel surface. Color measurement was carried out with regards to three coordinate values (L*, a*, b*), as established by Commission International de l’Eclairage (CIE). These parameters locate the color of an object in a three-dimensional color space. The L* axis quantifies the value or degree of lightness within a sample, and ranges from 0 (black) to 100 (white), whereas the a* plane represents the degree of red/green color (+a: red, -a: green) and the b plane corresponds with the degree of yellow/blue color (+ b: yellow, - b: blue) within the sample.

**Figure 1:**
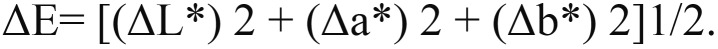
Formula.

The color measurements were made twice by 1 operator and the mean value was recorded for that specimen. The color difference between the four time points was measured using the following formula (Fig. 1):

-Cross-sectional Microhardness measurement

Enamel microhardness was determined using a micro Vickers hardness tester (Matsuzawa, model MHT2, Japan). The apparatus was used to create indentations on the enamel surface using a load of 100 g for 10 s. Three indentations were carried out on each specimen with a distance of 100 µm between them and the mean value was recorded as the Vickers hardness number (VHN) for that specimen.

-Statistical Analysis

The normal distribution of the data was confirmed by the Kolmogrov-Smirnov test. One-way analysis of variance (ANOVA) was run to compare the color change (∆E) obtained from the two measurements at T1 to T4 time points among the experimental groups, followed by Duncan post hoc test for pairwise comparisons. The intergroup differences in microhardness were also determined by ANOVA followed by Duncan test. The statistical analysis was performed by Statistical Package for the Social Sciences (SPSS; version 16.0, SPSS Inc, Chicago, Ill) and the level of significance was set at *p*<0.05.

## Results

 Table 1 displays the mean values, standard deviations (SD) and the results of the statistical analysis regarding the color change between different stages (ΔE) in the experimental groups. No significant difference was found in the color change between T1 and T2 (ΔET1-T2) and T3 and T4 (ΔET3-T4) time points among the study groups (*p*>0.05;  Table 1). The experimental groups, however, indicated statistically significant differences in the color change between T2 and T3 (ΔET2-T3) stages (*p*<0.05;  Table 1). Pairwise comparison by Duncan test revealed that ΔET2-T3 was significantly greater in group 4 (94% NaHCO3 + 1.5% H2O2 + CPP-ACPF) as compared to the other experimental groups (*p*<0.05). Furthermore, the values of ΔET2-T3 in groups 1 (94% NaHCO3), 2 (94% NaHCO3 + CPP-ACPF) and 3 (94% NaHCO3 + 1.5% H2O2) were comparable to each other (*p*>0.05), and all were significantly greater than that of the control group (*p*<0.05).

**Table 1:**
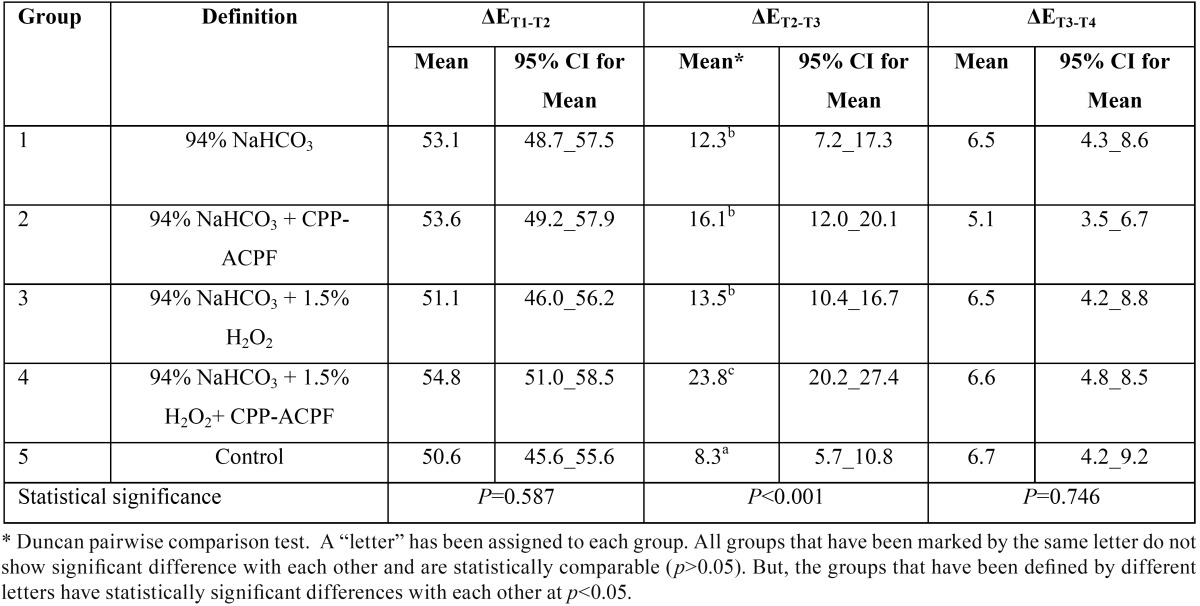
The means 95% confidence intervals (CI) and the results of the statistical analyses regarding color change values (ΔE) between different treatment stages in the study groups.

The results of the microhardness measurements are presented in  Table 2. The greatest microhardness was observed in group 4 (94% NaHCO3 + 1.5% H2O2 + CPP-ACPF) and the lowers one in group 5 (control). ANOVA displayed a statistically significant difference in microhardness among the experimental groups (*p*<0.001). Further analysis with Duncan test ( Table 2) revealed that the VHN in all the experimental groups were significantly greater than that of the control group (*p*<0.05). Furthermore, the VHN in group 4 was significantly higher than that of the group 1 (94% NaHCO3) specimens (*p*<0.05;  Table 2).

**Table 2:**
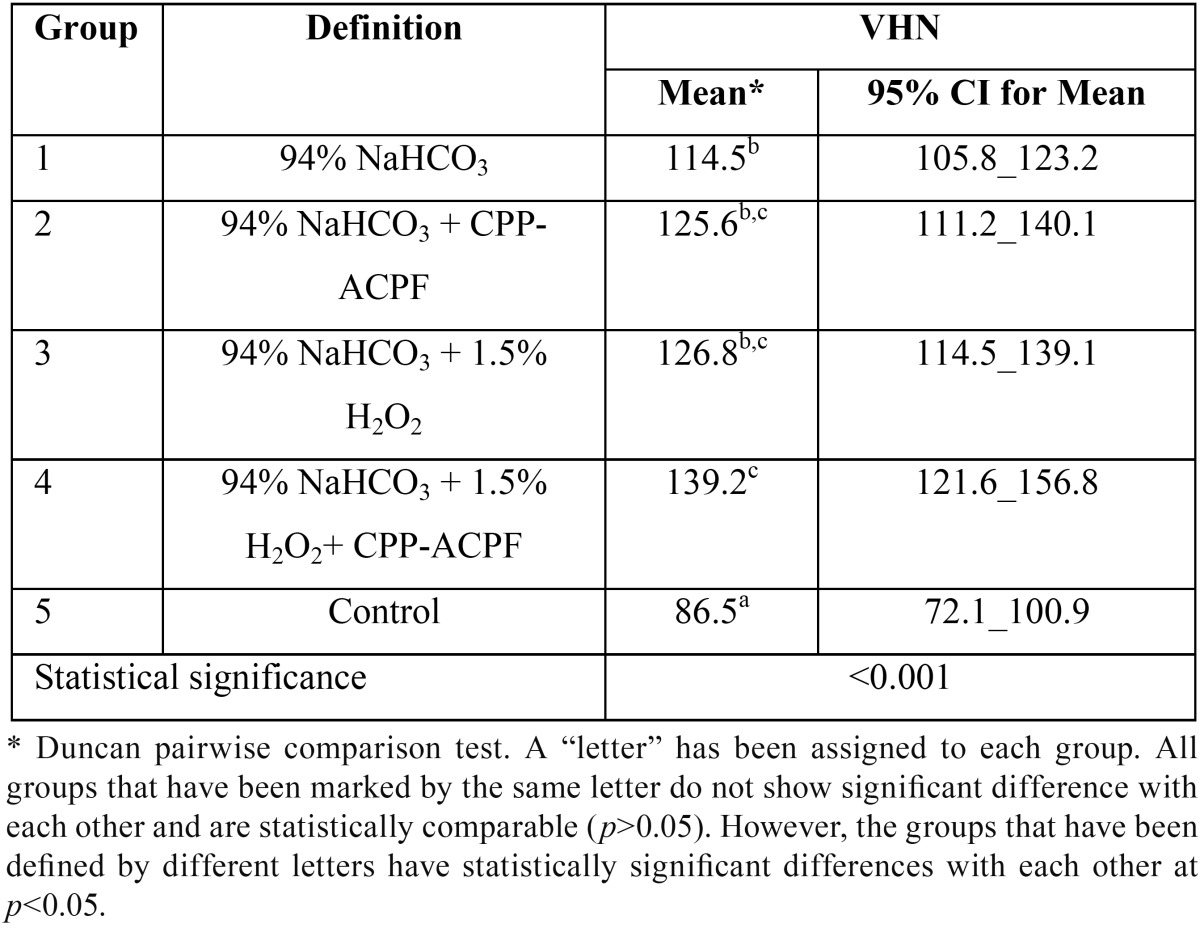
The means 95% confidence intervals (CI) and the results of the statistical analyses regarding Vickers Hardness Number (VHN) in the study groups.

## Discussion

This *in vitro* study investigated the effect of 94% NaHCO3 and 1.5% H2O2 in association with CPP-ACPF on color change and microhardness of teeth with extrinsic stains. The use of bovine incisors allowed the preparation of specimens with flat surfaces and dimensions consistent with the measuring window of the spectrophotometer. The Vita Easyshade spectrophotometer used in this study is a reliable, reproducible and quantitative device to assess alterations in tooth stain in both *in vitro* and *in vivo* conditions (21). The amount of ΔE represents the overall color change and values of at least 3.3 are known to be visually perceptible and clinically recognizable by human eyes (21).

In the present study, the degree of lightness decreased and the a, and b values increased in all groups following the artificial staining process. The total change between T1 and T2 stages (ΔET1-T2) was higher than 3.3, indicating that the staining procedure caused a clinically noticeable color change in all groups. There was no significant difference in ΔET1-T2 between the study groups, which was a prerequisite for a proper comparison of different treatments on discolored enamel.

After completing the 10-day whitening protocol (T3), it was revealed that the specimens treated with NaHCO3 (group 1), NaHCO3 + CPP-ACPF (group 2) or NaHCO3 + H2O2 (group 3) experienced comparable color improvement, which was significantly greater than that of the control group. The greatest whitening effect was observed in group 4 where both 1.5% H2O2 and CPP-ACPF were blended with NaHCO3. The values of ΔET2-T3 ranged from 8.3 in the control group to 23.8 in group 4. All the protocols were to some extent effective in tooth whitening, although none of them was capable to restore the original color of enamel.

Sodium bicarbonate (NaHCO3) or baking soda is commonly used in dentifrices because of its abrasivity, which leads to stain removal. Sodium bicarbonate is in the form of a white powder with an approximate Ph value of 8. The findings of this study indicated that sodium bicarbonate is effective for tooth whitening. Some authors believe that abrasives such as silica and sodium bicarbonate can eliminate extrinsic stains, but are not capable to clean deeper, intrinsic stains (22). Others reported an observable removal of intrinsic stains as a result of mechanical brushing with sodium bicarbonate-based dentifrices (1).

In the current study, we used an electric toothbrush after the application of whitening agents. It has been demonstrated that brushing is a necessary step for effective whitening of discolored teeth by sodium bicarbonate, as it can activate or accelerate the abrasitivity of this agent on tooth enamel, thereby enhancing its stain-removing ability (1). The concentration of sodium bicarbonate in this study was 94%. Kleber and Moore (1) reported that the ability of dentifrices for tooth whitening enhanced by increasing the concentration of sodium bicarbonate from 45% to 65% in a paste formulation; after that a plateau effect was observed, so that further increase in the concentration of sodium bicarbonate failed to enhance tooth whitening.

In the present study, a mild hydrogen peroxide agent was added to NaHCO3 in order to evaluate whether 1.5% H2O2 increases the whitening effect of the resulting mixture. It is believed that the decomposition of hydrogen peroxide leads to the formation of hydroxyl, perhydroxyl or superoxide anion radicals, which degrade high-molecular pigments to achromic low-molecular substances, thus producing the whitening effect (8,23,24). This study found no significant difference in removing tooth stain between the agent containing sodium bicarbonate and that containing sodium bicarbonate + 1.5% H2O2. The low concentration of H2O2 as used in this study may be the reason for its low stain-removing potential. Various concentrations of hydrogen peroxide can be used in whitening products and it is possible that significant effects appear at higher concentrations. The brushing time of 5 minutes (30 seconds for 10 times) in this study should be considered relatively short to represent the whitening potential of the experimental agents. Considering that an average adult brushes twice daily for at least 1 minute per time, the 5 minute-brushing occurs in less than 1 week, which is lower than that generally used with a whitening dentifrice. Kleber *et al.* (1) found that 30 minutes of brushing with sodium bicarbonate dentifrices lead to intrinsic stain removal and measurable tooth whitening. It is suggested that future studies evaluate the effect of increasing the duration and frequency of brushing with the experimental products in order to determine their effects on tooth whitening.

The outcomes of this study are in agreement with the results of Kleber *et al.* (1) who compared the whitening efficacy of various dentifrices and found that the inclusion of 1.5% H2O2 in the formulation containing 94% NaHCO3 provided no significant advantages over the use of 94% NaHCO3 dentifrice in removing tooth stains. They proposed that the concentration of 1.5% peroxide in the 94% NaHCO3 dentifrice was too low to exert a bleaching effect and suggested that at least 3% peroxide concentration should be present in bleaching agents for effective tooth whitening. In contrast, Kleber *et al.* (22) indicated that a baking soda dentifrice containing stabilized 1% hydrogen peroxide caused a significant decrease in yellow color (b*) of the teeth after 8 or more hours of topical treatment. This longer period of brushing compared to the 5-minute brushing in the present study may be the possible explanation for the conflicting results between these two studies.

In groups 2 and 4 of this study, the CPP-ACPF was added to the whitening agents. It is assumed that the use of CPP-ACP in association with the bleaching process prevents the adverse effects of whitening agents on tooth structure (9-15). Some authors believe that the mineral agents containing calcium and phosphate ions are more suitable than fluoride-containing agents to be used in bleaching products, because fluoride ions precipitate on the surface enamel and block further ion penetration into the subsurface lesion, thus limiting deeper remineralization (15,25,26). In the present study, the addition of CPP-ACPF to sodium bicarbonate did not reduce its whitening efficacy. Several studies also found that the bleaching potential of peroxides was not influenced by the application of CPP-ACP (9-11,13,15,27). In this study, we did not assess the net effect of CPP-ACP on tooth color. Interestingly, de Vasconcelos *et al.* (27) indicated that the gel containing “CPP-ACP” alone was effective in removing tooth stains. They proposed that the remineralizing action of CPP-ACP leads to an increase in luster and translucency of enamel and so inducing small improvement in tooth color (27).

In this study, the group 4 in which CPP-ACPF paste was blended with the whitening agents displayed the highest microhardness value among the study groups. The amount of microhardness in group 4 was significantly greater than that of the group 1 (94% NaHCO3) and 5 (control), but comparable with groups 2 (94% NaHCO3 + CPP-ACPF) and 3 (94% NaHCO3 + 1.5% H2O2). Likewise, in a study conducted by Bayrak *et al.* (14), the groups treated with daily application of CPP-ACP or CPP-ACPF pastes throughout the bleaching period showed significant increases in enamel microhardness following treatment. Cunha *et al.* (15) indicated that the use of CPP-ACP before/after the bleaching protocol was capable to prevent the adverse effects on roughness and hardness of bovine enamel. Although the present study did not evaluate the percentage of alteration in enamel microhardness after the whitening process, the reduction in mineral properties of enamel and dentin after the use of bleaching products has been reported in several investigations (4,8,28,29). The present study revealed that the addition of CPP-ACPF to sodium bicarbonate and 1.5% H2O2 can lead to higher mineral content at the end of the bleaching process compared to that observed in the sodium bicarbonate or control groups.

In a recent study, Singh *et al.* (16) indicated that treatment of bleached tooth surfaces with remineralizing agents such as CPP-ACP or fluoride resulted in less stain absorption and more color stability following exposure to a tea solution. However, in the present study, the addition of CPP-ACPF to NaHCO3 did not cause any significant reduction in stain absorption after the second staining period of 10 minutes. The difference in the composition of whitening agents and the mode of CPP-ACPF application on the enamel surface may be the reason for the contradictory outcomes of this study and those of Singh *et al.* (16).

Most patients undergoing fixed orthodontic therapy suffer from color alterations on their teeth a few days after starting treatment. The enamel discoloration affects the esthetics of the dentition and the patients are usually willing to remove them during orthodontic treatment, not waiting until the end of the therapy. The present study indicated that the use of a mild whitening agent containing 94% NaHCO3, 1.5% H2O2 and CPP-ACPF in association with tooth brushing can eliminate superficial stains and help the patients attaining whiter teeth during therapy. Since most patients show localized or generalized areas of demineralization after placement of fixed appliances, the use of this whitening agent can not only increase the bleaching efficacy and minimize the adverse effects of bleaching products, but also can help enamel remineralization. Further clinical studies with large sample sizes are warranted to investigate the efficacy of this mixture on whitening of teeth with extrinsic stains and elucidate its possible benefit in reducing mineral loss, tooth sensitivity and further stain absorption. In addition, it is suggested that future studies on the use of NaHCO3 assess the efficacy of longer periods of mechanical abrasion and higher concentrations of H2O2 in association with this abrasive agent.

## Conclusions

Within the limitations of the present study, the following conclusion could be drawn:

The specimens treated with a combination of 94% NaHCO3, 1.5% H2O2 and CPP-ACPF showed the greatest color change and microhardness of enamel at the end of the whitening process. Therefore, the application of a cream containing NaHCO3, 1.5% H2O2 and CPP-ACPF is suggested in the clinical situation for patients with extrinsic stain especially those who also show demineralized enamel.
